# Predominance of triple wild-type and IGF2R mutations in mucosal melanomas

**DOI:** 10.1186/s12885-018-4977-2

**Published:** 2018-10-30

**Authors:** Yuuki Iida, Matthew P. Salomon, Keisuke Hata, Kevin Tran, Shuichi Ohe, Chester F. Griffiths, Sandy C. Hsu, Nellie Nelson, Dave S. B. Hoon

**Affiliations:** 1 Department of Translational Molecular Medicine, Division of Molecular Oncology, John Wayne Cancer Institute at Providence Saint John’s Health Center, Santa Monica, CA 90404 USA; 2 Brain Tumor Center, Providence Saint John’s Health Center, Santa Monica, CA USA; 3 John Wayne Cancer Institute Genome Sequencing Center, John Wayne Cancer Institute at Providence Saint John’s Health Center, Santa Monica, CA USA

**Keywords:** Mucosal melanoma, Triple wild-type, Tobacco exposure, IGF2R, DCC

## Abstract

**Background:**

Primary mucosal melanoma (MM) is a rare subtype of melanoma that arises from melanocytes in the mucosa. MM has not been well profiled for mutations and its etiology is not well understood, rendering current treatment strategies unsuccessful. Hence, we investigated mutational landscape for MM to understand its etiology and to clarify mutations that are potentially relevant for MM treatment.

**Methods:**

Forty one MM and 48 cutaneous melanoma (CM) tissues were profiled for mutations using targeted deep next-generation sequencing (NGS) for 89 cancer-related genes. A total of 997 mutations within exons were analyzed for their mutational spectrum and prevalence of mutation, and 685 non-synonymous variants were investigated to identify mutations in individual genes and pathways. PD-L1 expression from 21 MM and 18 CM were assessed by immunohistochemistry.

**Results:**

Mutational spectrum analysis revealed a lower frequency of UV-induced DNA damage in MM than in CM (*p* = 0.001), while tobacco exposure was indicated as a potential etiologic factor for MM. In accordance with low UV damage signatures, MM demonstrated an overall lower number of mutations compared to CM (6.5 mutations/Mb vs 14.8 mutations/Mb, *p* = 0.001), and less PD-L1 expression (*p* = 0.003). Compared to CM, which showed frequent mutations in known driver genes (*BRAF* 50.0%*, NRAS* 29.2%), MM displayed lower mutation frequencies (*BRAF;* 12.2%, *p* < 0.001, *NRAS*; 17.1%), and was significantly more enriched for triple wild-type (no mutations in *BRAF*, *RAS*, or *NF1*, 70.7% vs 25.0%, *p* < 0.001), *IGF2R* mutation (31.7% vs 6.3%, *p* = 0.002), and *KIT* mutation (9.8% vs 0%, *p* = 0.042). Of clinical relevance, presence of *DCC* mutations was significantly associated with poorer overall survival in MM (log-rank test, *p* = 0.02). Furthermore, mutational spectrum analysis distinguished primary anorectal MM from CM metastasized to the bowel (spectrum analysis *p* < 0.001, number of mutations *p* = 0.002).

**Conclusions:**

These findings demonstrated a potential etiologic factor and driver mutation for MM and strongly suggested that MM initiation or progression involves distinct molecular-mechanisms from CM. This study also identified mutational signatures that are clinically relevant for MM treatment.

**Electronic supplementary material:**

The online version of this article (10.1186/s12885-018-4977-2) contains supplementary material, which is available to authorized users.

## Background

Primary mucosal melanoma (MM) is a rare subtype of melanoma which accounts for approximately 1% of melanoma and arises from melanocytes in mucosal tissue of different anatomical sites, such as the head & neck, gastrointestinal tract, or genitourinary tracts [1–3]. Compared to cutaneous melanoma (CM), MM is relatively asymptomatic or lacks early clinical visibility, which is important for early detection of CM, thus it is often diagnosed at more advanced stages and therefore, exhibiting poor prognosis. The treatment of MM remains subjective because of the rareness of the cancer and lack of randomized controlled trials [1–3]. Epidemiologic studies have indicated potential risk factors for MM, such as tobacco exposure, HIV infection, or chronic inflammation [4], however precise roles of these factors on MM remain unknown.

In a recent study [5], CM was stratified into four molecular subtypes: *BRAF* mutated, *RAS* mutated (*NRAS*/*KRAS*/*HRAS*), *NF1* mutated (a regulator of RAS pathway [6]), and triple wild-type (Triple-WT, a subgroup that lacks above mutations) using the Cancer Genome Atlas (TCGA) database. Molecular targeted therapies, such as BRAF inhibitors or MEK inhibitors, are applicable for CM treatment based on these genetic subtypes [7]. However, mutational patterns in MM have been profiled for only a few genes, such as *BRAF*, *NRAS*, and *KIT*, which are mostly targeted for specific hotspots or limited regions within the genes [1, 3, 8]. Our knowledge about the cancer-related gene mutations in MM, particularly in all exonic regions, is still limited and warrants further investigation into the mutational landscape to understand the etiology of the disease and better treatment strategies for MM.

To date, the majority of genomic studies aimed to identify somatic mutations in melanoma have focused mainly on CM and have only include a small number of MM samples (i.e. [9, 10]). Therefore, to better investigate the etiology of MM and to clarify mutations that are potentially relevant for MM treatment, we utilized targeted next-generation sequencing (NGS), which enabled us to screen all exons on multiple cancer-related genes with high coverage [11–14] in a large collection of MM. Here, we analyzed the mutational landscape between 41 MM and 48 CM specimens using our custom panel of 89 genes frequently mutated in cancer.

## Methods

### Patients and specimen collection

The study involved clinically and surgically defined tumors that comprised of 41 MM and 48 CM from the John Wayne Cancer Institute (JWCI) tissue bank archive. All surgeries were performed at Saint John’s Health Center (SJHC), and specimens were identified by experienced melanoma surgical pathologists at SJHC, Dept. of Surgical Pathology. Detailed characteristics of specimens are shown in Additional file 1: Table S1. In MM patients, metastases from CM were ruled out as they had no medical history or evidence of CM or uveal melanoma. This study followed the principles in the Declaration of Helsinki. All human specimens and clinical information for this study, including informed consent, were obtained according to the protocol guidelines approved by the SJHC/JWCI Western Institutional Review Board.

### DNA extraction

Frozen tissues (*n* = 62) were homogenized with a sonicator and filtered using QIAshredder (QIAGEN, Valencia, CA), and DNA was extracted using the ZR-Duet DNA/RNA MiniPrep (Zymo Research, Irvine, CA), according to the manufacturer’s protocol. DNA extraction from formalin-fixed paraffin-embedded (FFPE) specimens (*n* = 27) was performed using ZR FFPE DNA MiniPrep (Zymo Research), as previously described [15]. DNA was quantified using UV spectrophotometer (BioTek, Winooski, VT) and Quant-iT PicoGreen dsDNA Assay Kit (ThermoFisher Scientific, Carlsbad, CA). For specimens contaminated with strong melanin content, OneStep PCR Inhibitor Removal Kit (Zymo research) was used for melanin removal. For tumor purity, we assessed frozen tissues that were dissected by a surgical pathologist from the original tumor surgery whereby a representative tissue was made into a FFPE tumor block. The majority (> 90%) of the cells in the frozen tissues were melanoma cells. For FFPE tumor block analysis, we assessed H&E stained slides and performed micro-dissection of melanoma tumor cells.

### Custom target enrichment and NGS

An Agilent Haloplex custom target enrichment kit that captured all exons in 89 genes related to melanoma and tumors from mucous membranes [5, 16–19] was designed using the Agilent SureDesign software (Agilent Technologies, Santa Clara, CA). The genes on the panel are listed in Additional file 2: Table S2. Target-enriched libraries were constructed from genomic DNA (3 μg), following an instruction from HaloPlex Target Enrichment for Illumina Kit (Agilent Technologies) [13]. Only library fragments within 175 to 625 bp were considered for the final quantification, normalization, and pooling. The final multiplexed Haloplex custom target library pool was sequenced on the Illumina HiSeq 2500 (Illumina, San Diego, CA) on rapid mode using paired-end 100 bp reads. Overall, the panel showed high coverage rates (median 349x) for all the target regions.

### Variant calling and data analysis

Raw genomic sequence reads were mapped to the 1000 Genomes (b37) built of the human genome reference using BWA-MEM (version 0.7.5a) with default settings [20]. The resulting alignments were further processed using GATK (version 2.8–1) following the GATK Best Practices recommendations [21–23]. Single nucleotide variants were identified using MuTect version 1.1.4 [24]. MuTect was run using default parameters in the High Confidence mode along with dbSNP (version 137) and COSMIC (version 67) databases on tumor only samples. Only SNVs that were classified as “KEEP” by MuTect were used for downstream analysis. Genomic annotations were performed using the ANNOVAR annotation pipeline [25]. In order to remove potential germline variants from our set of variants, we further filtered the variants using SnpSift [26]. Only variants that had a population frequency below 1%, mutation allele frequency > 10%, and coverage of >20X were used for further analysis. The final mutation call set had a mean depth of coverage of ~274X. Mutation spectrum and signatures analyses were done using the Bioconductor packages SomaticSignatures [27] and deconstructSigs [28], respectively. A total of 997 mutations that were located within exonic regions were analyzed for mutation spectrum and prevalence of mutations, and 685 non-synonymous mutations were investigated for identification of MM associated genes and pathways. MAPK pathway, one of the most biologically and clinically important pathways in melanoma [5], was considered to be mutated when there was at least one non-synonymous mutation in *BRAF*, *HRAS*, *KRAS*, *MAPK2K1*, *NF1* or *NRAS*.

### Immunohistochemistry (IHC)

IHC was performed as previously described [29, 30], using anti-PD-L1 rabbit monoclonal antibody (1:100 dilution, #ab205921; Abcam). After the antigen retrieval step, the sections were incubated in 10 g/L trichloroisocyanuric acid (176,125; Sigma-Aldrich) solution for 30 min at room temperature to bleach the melanin [31]. Photographs were obtained using a Nikon Eclipse Ti microscope and NIS elements software (Nikon). Total of 39 specimens (21 MM and 18 CM) were available for immunohistochemistry. The expression of PD-L1 was quantified using an H score system, which considers both the intensity and percentage of positive cells [32]. Intensity of PD-L1 on tumor cell membrane was determined between 0 (no staining), 1 (weak), 2 (moderate), and 3 (strong). The score was calculated using the following formula; 1 x (% of 1+ cells) + 2 x (% of 2+ cells) + 3 x (% of 3+ cells) [32] .

### Data access

Raw genomic sequence data obtained in this study can be accessed from NCBI SRA under Bioproject number PRJNA379027.

### Pan cancer mutation analysis

TCGA mutation frequency data was downloaded using the RTCGAToolbox Bioconductor package for run date “20,150,821” [33].

### Statistical analysis

Continuous variables were assessed using Student’s *t* test or Wilcoxon rank-sum test, and categorical variables were assessed using *χ*^2^ test or Fisher’s exact tests. FDR corrected *p*-value < 0.05 was considered to perform multiple test for 96 substitutions. Overall survival (OS) was analyzed based on the time since being diagnosed with melanoma using the Kaplan-Meier method and log-rank test. All statistical analyses were performed with JMP, version 11.0 (SAS Institute Inc., Cary, NC) or R (https://www.R-project.org), and a two-sided *p*-value < 0.05 was regarded as statistically significant.

## Results

### Mutation spectrum analysis revealed a potential risk factor for MM

**Fig. 1:**
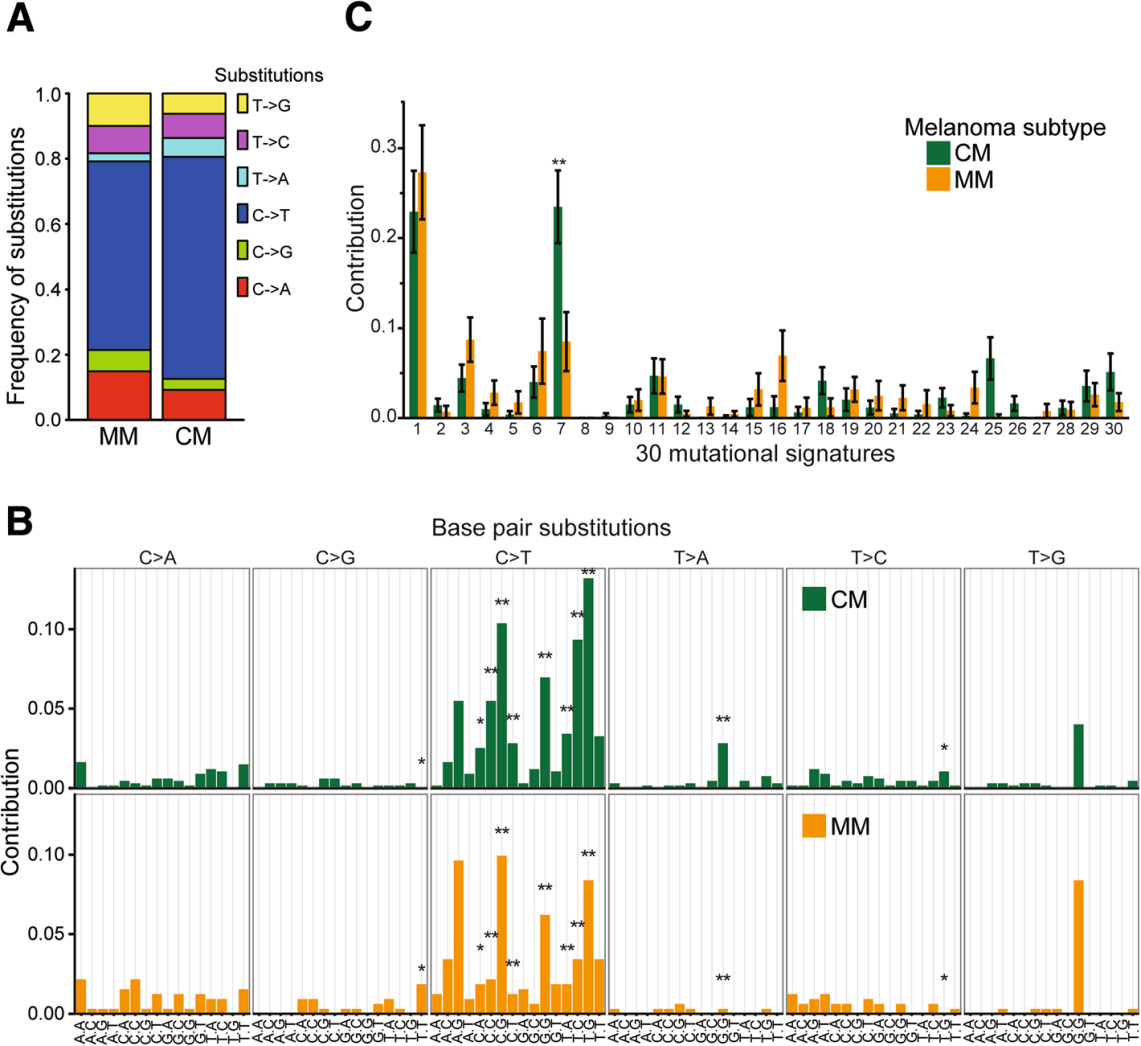
Mutational spectrum analysis for MM and CM. MM presented a distinct mutational spectrum compared to CM. **a** Bar plots showing the frequency of six substitutions in MM (*n* = 41) and CM (*n* = 48). **b** & **c** Bar plots showing the frequency of (**b**) 96 substitutions and (**c**) 30 mutational signatures from COSMIC in MM and CM. Error bars represent means ± standard deviation (*p < 0.05, **p < 0.01)

UV exposure is a major mutagenic factor that drives malignant transformation of melanocytes into CM [34]. However, MM is generally not exposed to UV due to its anatomical locations [1, 3, 8]. Epidemiologic studies have indicated potential risk factors for MM, including tobacco use for oral MM [4, 35]; however, no definitive risk factor has been identified. To investigate the involvement of UV or tobacco exposure in MM, we performed a mutational spectrum analysis for a total of 997 mutations on exons in 89 genes. Within six different substitutions, C > T substitutions, were the most predominant substitution type in both MM and CM (Fig. 1a). Importantly, the C > T substitutions were significantly less in MM (57.8%) than CM (64.8%, Fig. 1a, *χ*^2^ test, *p* = 0.002). Despite the lower frequency of C > T substitutions in MM, they were still the most predominant substitution type in MM. Other factors, such as aging, induce C > T substitutions and therefore are the most prevalent substitutions in many cancers [16, 36], including other mucosal origin tumors such as gastric adenocarcinoma and colorectal cancer (CRC) [16, 19] and are not highly specific to UV damage [16, 36].

To further analyze the mutational spectrum in MM, information on the nucleotides immediately 5′ and 3′ to each mutated base (i.e. tri-nucleotide context) were incorporated into our analysis, making 96 possible substitutions [36, 37]. Eleven substitutions types, mostly C > T substitutions, significantly differed between MM and CM (Fig. 1b, *t*-test, FDR corrected *p* < 0.05). We categorized these 96 substitutions into 30 different signatures defined by the COSMIC (Catalogue of somatic mutations in cancer, http://cancer.sanger.ac.uk/cosmic/signatures, see methods) database. These 30 different signatures propose specific etiologies or mutational mechanisms that lead to specific signatures. As expected, the UV damage signature (signature 7) was less enriched in MM (Fig. 1c, Wilcoxon, *p* = 0.001), consisted with the C > T mutational spectrum analysis above. Besides the UV damage signature, eight MM specimens (19.5%) presented signatures for tobacco exposure (signature 4 and 29). Of the eight MM that displayed the tobacco related mutation signature, 5 were head & neck (29% of 17 head & neck cases), 1 was genital (10%), and 2 were anorectal (14%). While patient smoking history was not available for the individuals included in this study and while the precise mechanisms of how tobacco exposure might drive MM remains unknown, this mutational spectrum analysis suggests smoking is a potential pathogenic factor in MM.

### Tumor mutation burden and PD-L1 expression are less in MM

**Fig. 2:**
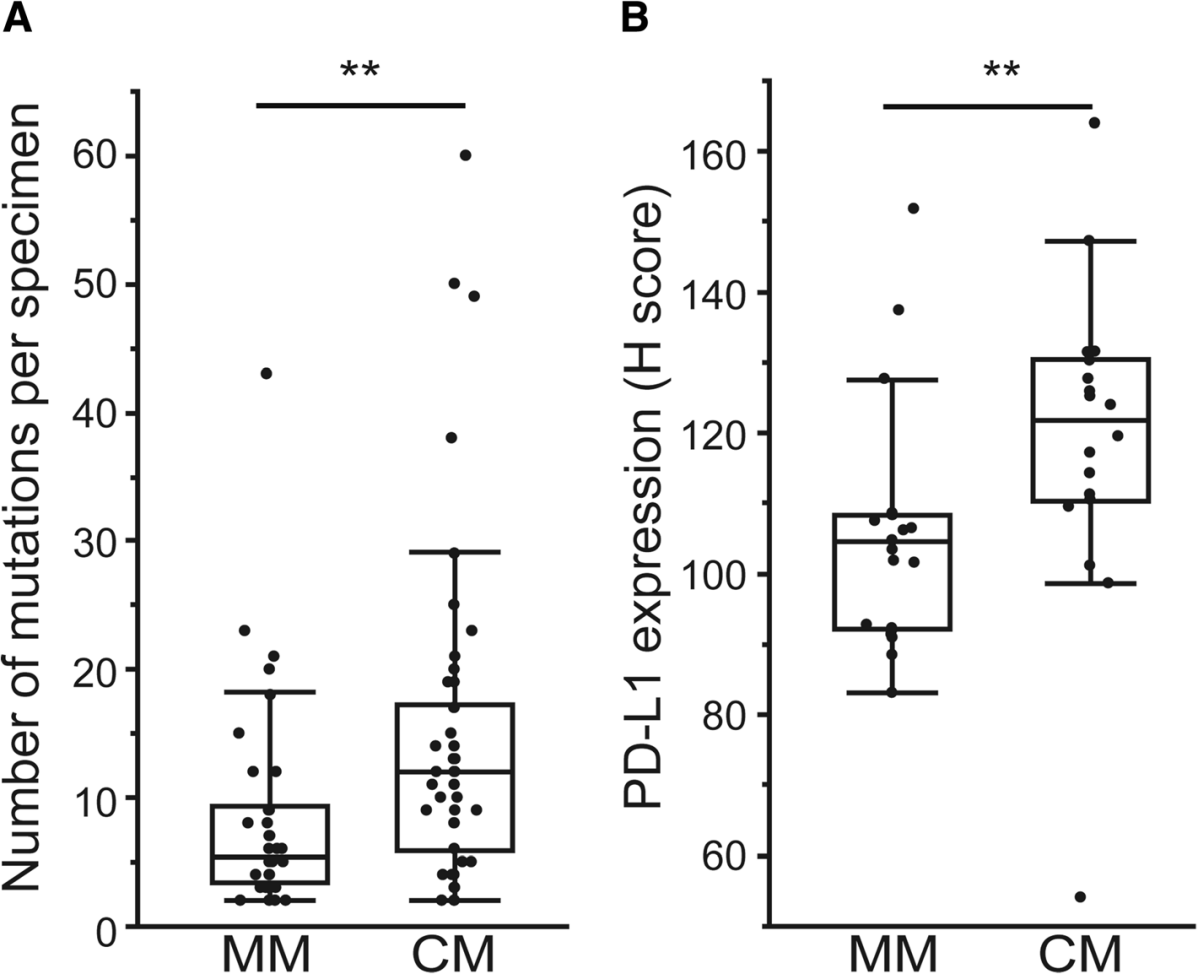
Tumor mutation burden and PD-L1 expression in MM and CM. MM presented lower tumor mutation burden and PD-L1 expression compared to CM. **a** Number of mutations per sample was assessed by mutational spectrum analysis. Box plots showing the number of mutations per specimen in MM (*n* = 41) and CM (*n* = 48). **b** PD-L1 expression was analyzed for MM and CM by immunohistochemistry. Box plots showing the PD-L1 expression (H score) in MM (*n* = 21) and CM (*n* = 18) (**p < 0.01)

Due to lower exposure of mutagenic UV light in MM (Fig. 1a, c), we speculated that mutations are less prevalent in MM than CM. CM, which exhibits the highest tumor mutation burden among different cancer types [36], demonstrated 14.8 mutations/Mb (median, 11.5 mutations per sample) in our cohort, while the number of mutations was significantly less in MM (median 6.5 mutations/Mb (5.0 mutations per sample), Fig. 2a, Wilcoxon, *p* = 0.001). We further investigated PD-L1 expression in both types, since PD-L1 expression is positively associated with tumor mutation burden [38] and predicts clinical benefit from immunotherapies in different tumors, including CM [39–41]. In accordance with low tumor mutation burden, PD-L1 expression was significantly less in MM compared to CM (Fig. 2b, Wilcoxon, *p* = 0.003), suggesting that immune checkpoint inhibitors, such as nivolumab or pembrolizumab, should be carefully considered to apply to MM patients.

### *IGF2R* and *KIT* mutations are prevalent in MM

To investigate mutations of potential driver genes in MM, we analyzed 685 non-synonymous variants and evaluated prevalence of mutations in each gene. Previous studies have demonstrated that CM has a high prevalence of driver gene mutations, such as *BRAF* mutations or *NRAS* mutations [5, 7], while these driver mutations are less frequent in MM [3, 8]. In our cohort, mutations in *BRAF*, a major driver gene in CM [5], was significantly less frequent in MM than CM (12.2% vs 50%, Tables 1, 2, Fisher’s exact test, *p* < 0.001). Within *BRAF* mutations, 75% and 87% of were located in the *BRAF*^V600^ hotspot mutation in our CM cohort and in the TCGA cohort [5], respectively. In contrast, the *BRAF*^V600^ hotspot mutation was not present in MM. Frequency of *NRAS* mutations, another major driver mutation in CM, was also lower in MM (17.1%) than CM (29.2%) (Table 1). Moreover, other driver mutations (*KRAS* mutations 2.4%, *HRAS* mutations 2.4%, *TP53* mutations 7.3%) were not prevalent in MM.

**Table 1:** Frequently mutated genes in MM and CM

MM (*n* = 41)		CM (*n* = 48)	
GENE	%	GENE	%
IGF2R	31.7	FAT4	54.2
KMT2A	22.0	BRAF	50.0
ATM	17.1	DCC	41.7
NRAS	17.1	NRAS	29.2
NF1	14.6	KMT2A	25.0
TET2	14.6	ATM	25.0
ACTL6A	12.2	NF1	20.8
APC	12.2	BRCA2	20.8
BRAF	12.2	MET	20.8
BRCA2	12.2	TSC2	18.8
DCC	12.2	ARID1A	18.8
TSC2	12.2	ATR	18.8
FAT4	9.8	MTOR	18.8
KIT	9.8	EPHA3	18.8
LRP5	9.8	FZD10	16.7
RET	9.8	TET2	14.6
TCF7L2	9.8	APC	14.6
		LTK	14.6

**Table 2:** Differentially mutated genes between MM and CM

GENE	MM % (*n* = 41)	CM % (*n* = 48)	*p*-value (Fisher’s exact tests)
IGF2R	31.7	6.3	0.002
KIT	9.8	0.0	0.042
BRAF	12.2	50.0	< 0.001
FAT4	9.8	54.2	< 0.001
DCC	12.2	41.7	0.002
EPHA3	2.4	18.8	0.018

**Fig. 3:**
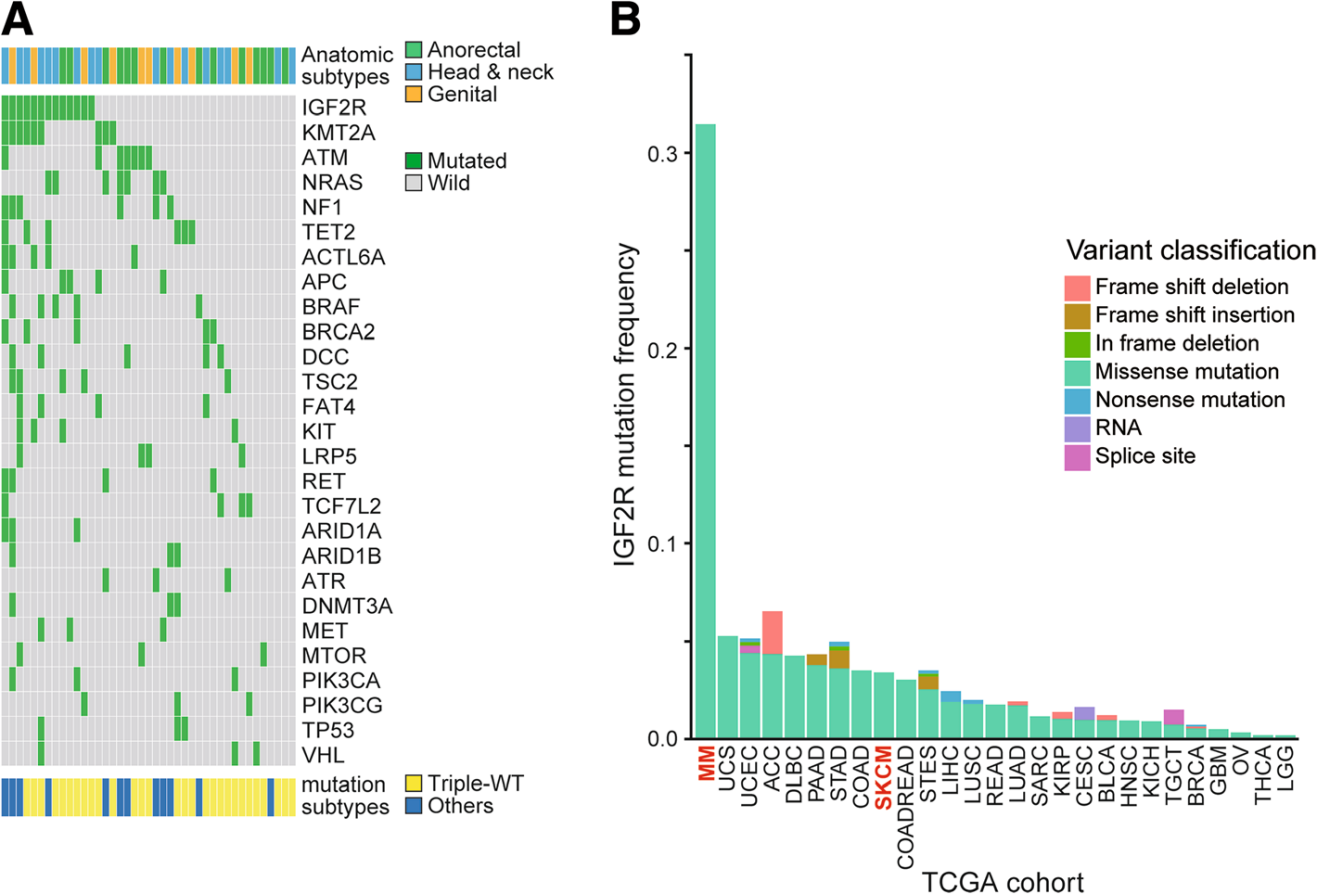
Landscape of mutations in MM. **a** A mutation status matrix was constructed from MM specimens. Frequently mutated genes along with anatomic subtypes (anorectal, head & neck or genital) and mutation subtype (Triple-WT or non-Triple-WT) were annotated for each specimen. **b** mutation frequency for *IGF2R* in different cancer types was investigated using TCGA database and our MM cohort. Bar plots showing frequency of *IGF2R* mutations along with the types of mutations. UCS: uterine carcinosarcoma, UCEC: uterine corpus endometrial carcinoma, ACC: adrenocortical carcinoma, DLBC: lymphoid neoplasm diffuse large B-cell lymphoma, PAAD: pancreatic adenocarcinoma, STAD: stomach adenocarcinoma, COAD: colon adenocarcinoma, SKCM: skin CM, COADREAD: colorectal adenocarcinoma, STES: stomach and esophageal carcinoma, LIHC: liver hepatocellular carcinoma, LUSC: lung squamous cell carcinoma, READ: rectum adenocarcinoma, LUAD: lung adenocarcinoma, SARC: sarcoma, KIRP: kidney renal papillary cell carcinoma, CESC: cervical squamous cell carcinoma and endocervical adenocarcinoma, BLCA: bladder urothelial carcinoma, HNSC: head and neck squamous cell carcinoma, KICH: kidney chromophobe, TGCT: testicular germ cell tumors, BRCA: breast invasive carcinoma, GBM: glioblastoma multiforme, OV: ovarian serous cystadenocarcinoma, THCA: thyroid carcinoma, and LGG: brain lower grade glioma

Although MM exhibited less mutations in common driver genes for CM, we identified mutations in two genes that were significantly more frequent in MM than CM (Table 2). Mutations in *IGF2R*, which is involved in the insulin-like growth factor (IGF) pathway [42], was the most frequent mutation in MM (31.7%) and was significantly more prevalent than CM (6.3%, Table 2, Fisher’s exact test, *p* = 0.002, Fig. 3a). Notably, the frequency of *IGF2R* mutations were higher than other cancer types from TCGA database (Fig. 3b). In accordance with previous reports [3, 8, 43], *KIT* mutations were also significantly more frequent in MM (9.8% vs 0%, Fisher’s exact test, *p* = 0.042) (Table 2). Overall, MM showed mutated genes that are distinct from CM and was significantly more frequent in *IGF2R* and *KIT* mutations.

### Predominance of triple-WT in MM

We further classified the 89 samples into the four molecular subtypes, *BRAF* mutated (47% in melanoma TCGA), *RAS* mutated (*NRAS*/*KRAS*/*HRAS* mutations, 29%), *NF1* mutated (9%), and Triple-WT (subgroup lacking above mutations, 15%), proposed by the recent TCGA melanoma cohort [5]. Notably, MM showed significantly higher prevalence of the Triple-WT subtype than CM (70.7% vs 25.0%, Fisher’s exact test, *p* < 0.001). In accordance with the high prevalence of Triple-WT in MM, mutations in the MAPK pathway (mutations in any of the following genes; *BRAF*, *HRAS*, *KRAS*, *MAPK2K1*, *NF1* or *NRAS*) was significantly less frequent in MM than CM (36.6% vs 81.3%, Fisher’s exact test, *p* < 0.001). To further characterize the Triple-WT in MM, we compared Triple-WT (*n* = 29) and non-Triple-WT (*n* = 12) in MM. Despite the difference in driver gene mutations between Triple-WT and non-Triple-WT, there was no significant difference in their UV damage signatures (C > T substitutions, *χ*^2^ test, *p* = 0.35; signature 7, Wilcoxon, *p* = 0.39). Accordingly, the number of mutations were similar in both types (median 6.5 mutations/MB (5.0 mutations per sample) for both types, Wilcoxon, *p* = 0.78). Furthermore, there was no significant difference in overall survival (OS; log rank test, *p* = 0.43). Despite the similarity in the above mutational landscapes between Triple-WT and non-Triple-WT in MM, prevalence of Triple-WT was different between anatomical subtypes of MM. Dividing the MM into three major anatomic groups, genital (*n* = 10), head & neck (*n* = 17), and anorectal (*n* = 14) melanoma, frequencies of Triple-WT were 90.0%, 70.6% and 57.1%, respectively, suggesting distinct genetic background underlining MM anatomic subtypes.

### *DCC* mutation is a potential prognostic marker for MM

**Fig. 4:**
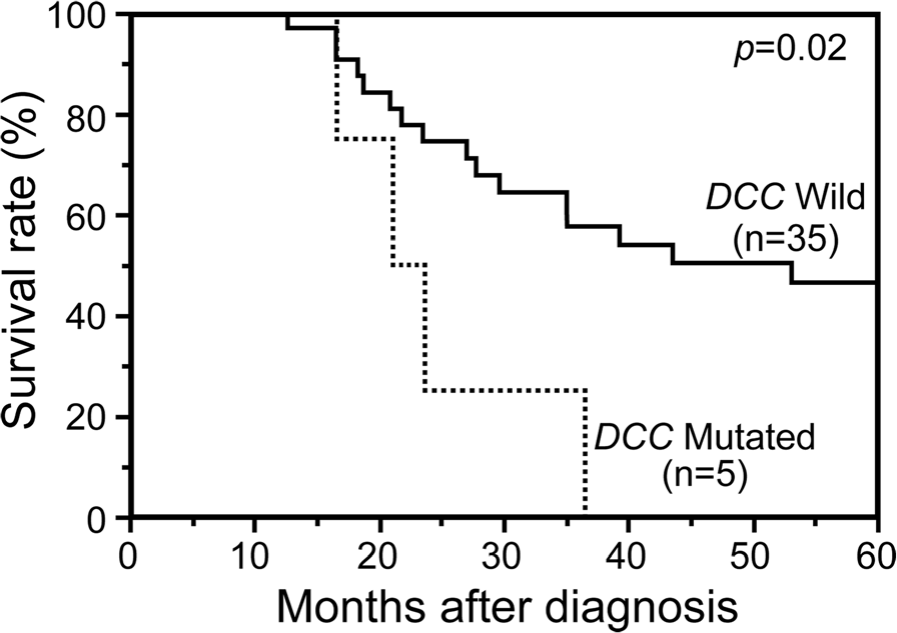
Potential prognostic marker in MM. Mutation status for each gene was investigated for survival analysis. Kaplan-Meier curves showing OS between *DCC* wild type or mutated specimens in MM. Significance of log rank is shown

We further investigated any potential prognostic markers for MM. *DCC* mutations were observed only in five patients from MM (12.2%) and was less prevalent in MM than in CM (Table 2). However, the presence of *DCC* mutations was significantly associated with poor OS in MM (Fig. 4, log-rank test, *p* = 0.02). In contrast, although *DCC* mutations were significantly more frequent in CM than MM (41.7% vs 12.2%), *DCC* mutations in CM was not associated with patients’ prognosis (log-rank test, *p* = 0.87). These results suggest the potential of *DCC* mutations as a potential prognostic marker in MM.

### Mutational spectrum analysis distinguishes primary anorectal MM from CM metastasized to the bowel

**Fig. 5:**
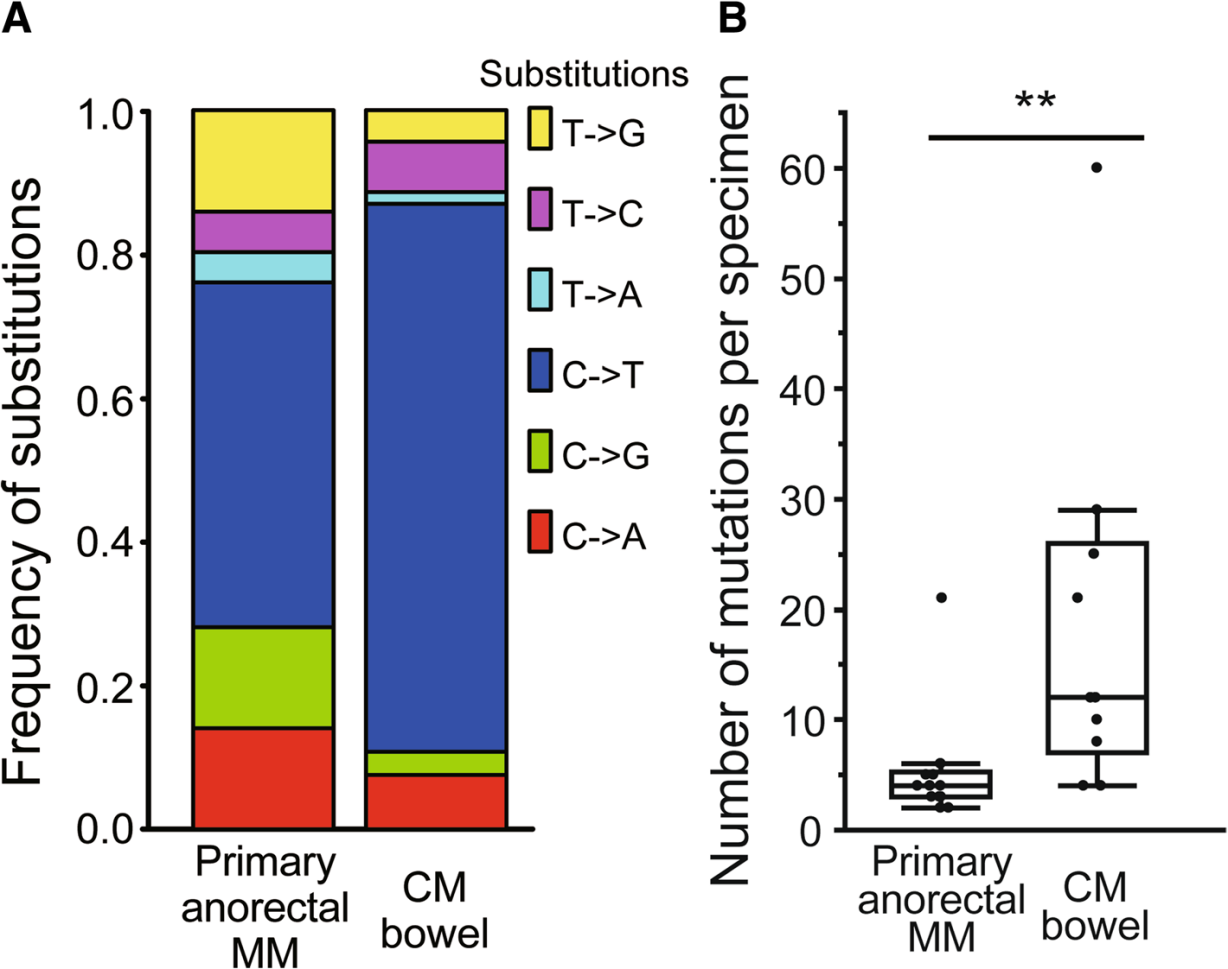
Mutational spectrum analysis distinguishes primary anorectal MM from CM metastasized to the bowel. Mutational spectrum and mutation prevalence were investigated for primary anorectal MM (*n* = 14) and CM metastasized to the bowel (CM bowel, *n* = 10). **a** Bar plots showing the frequency of six substitutions in primary anorectal MM and CM bowel. **b** Box plots showing the number of mutations per specimen in primary anorectal MM and CM bowel (**p < 0.01)

Melanomas in the anorectal region, primary anorectal MM or CM that has metastasized to the bowel, are rare diseases [44], and distinguishing these two types is occasionally challenging due to similarity in their anatomical sites [45]. Definite diagnosis for these types is clinically important, since CM has been well investigated for different treatment strategies, such as molecular targeted therapies or immunotherapies [7, 40, 41]. MM demonstrated a distinct mutational spectrum and tumor mutation burden from CM (Fig. 1a, c), thus we speculated that targeted NGS would enable us to distinguish between these types. Similar to Fig. 1a, CM metastasized to the bowel (*n* = 10) displayed significantly higher prevalence of C > T substitutions compared to anorectal MM (*n* = 14) (Fig. 5a, 76.2% vs 47.9%, χ^2^ test, *p* < 0.001). Accordingly, mutations were significantly more frequent in CM metastasized to the bowel than in anorectal MM (Fig. 5b, median 15.5 mutations/Mb (12 mutations per sample) vs 5.2 mutations/Mb (4 mutations per sample), Wilcoxon test, *p* = 0.002). Overall, mutational analyses were able to distinguish primary anorectal MM from CM metastasized to the bowel.

## Discussion

This study identified distinct mutational landscapes between MM and CM, particularly the signature for UV-induced DNA damage, and revealed that common driver gene mutations for CM were less frequent in MM. Although malignant transformation of melanocytes into CM is highly related to UV damage, and *BRAF* or *NRAS* mutations are involved in the progression of CM [5, 34], our study strongly suggested that MM likely has distinct mechanisms involved in its initiation and progression pathways. This is presumably from tobacco exposure or mutation in *IGF2R* and *KIT*, and thus MM may require different treatment strategies from CM.

Triple-WT comprises 15% of CM, as was previously identified in the melanoma TCGA cohort [5]. This subtype lacks hotspot *BRAF*, *RAS*, or *NF1* mutations which are important driver genes for CM. Triple-WT in CM has unique molecular characteristics such as amplifications of KIT, PDGFRA, VEGFR2, MDM2, or TERT, as well as an enrichment of complex structural rearrangements like fusion of driver genes [5]. Compared to CM, MM was highly associated with Triple-WT (70.7%). Despite the distinct characteristics between Triple-WT and non-Triple-WT, we observed no significant difference in mutational spectrum, tumor mutation burden, or prognosis between Triple-WT and non-Triple-WT in MM. Further genetic and epigenetic landscapes need to be elucidated to comprehensively investigate biological and clinical relevance of Triple-WT in MM.

In addition to Triple-WT, our study provides several important implications for the treatment of MM, particularly related to mutations in *IGF2R* and *DCC* genes. MM was significantly associated with *IGF2R* mutations, which are relatively low in other cancer types from TCGA database (Fig. 3b), indicating the unique genetic background of MM. Notably, none of the *IGF2R* mutations were recurrent at a single locus, signifying the importance of screening all exons within the gene panel. IGF2R is a multifunctional receptor and is involved in the IGF pathway [42]. The IGF pathway is triggered by IGF ligands (insulin, IGF1, or IGF2) binding to their receptors (insulin receptor or IGF1R) [46]. Stimulation of the pathway contributes to carcinogenesis or tumor progression in different tumors, including melanoma [40, 46–48]. IGF2R also has a high affinity for IGF ligands, particularly IGF2; however, the receptor lacks an intracellular tyrosine kinase domain that is essential for the activation of the IGF pathway, thus, the receptor acts as a “decoy” of the IGF pathway and is recognized as a tumor suppressor gene [42, 46, 47]. Although deregulation of IGF pathway through amplification or overexpression of IGF2 is involved in another mucosal-origin tumor, CRC [17], clinical relevance of *IGF2R* mutations is still controversial [42]. Inhibitors targeting the IGF pathway, such as anti-IGF1R antibodies in ongoing clinical trials [49], are potential candidates for MM treatment.

*DCC* mutations is another candidate that is potentially relevant for MM treatment. *DCC* codes netrin-1 receptor, which prevents apoptosis by binding to netrin-1. However, netrin-1 shortage induces cleavage of DCC at its intracellular domain and promotes apoptosis; thus, *DCC* is considered as a tumor suppressor [50–52]. Although the significance of *DCC* in melanoma remains unknown, mutations could lead to deregulation of DCC, possibly affecting the prognosis of MM. Interestingly, *DCC* mutations were more prevalent in CM; however, it was significantly associated with poor prognosis in MM, but not in CM. These results imply distinct biological significance of *DCC* mutations in MM and CM.

In addition to molecular targeted therapies applicable for individual mutations, immune checkpoint inhibitors that target CTLA-4 (cytotoxic T lymphocyte antigen 4), PD-1 (Programmed death 1), or PD-L1 (Programmed death ligand 1) demonstrates great promise for treatment of different tumors, including CM [39–41]. Particularly, drugs that block PD-1 (nivolumab or pembrolizumab) lead to significant improvement in CM treatment [40, 41]. MM also demonstrates higher response to nivolumab compared to ipilimumab (CTLA-4 inhibitor) [53]. PD-L1 expression, which is positively associated with tumor mutation burden [38], is clinically important as it predicts a better response to anti-PD-1 therapy in CM [40, 41]. In this study, MM demonstrated significantly lower tumor mutation burden compared to CM, and accordingly, lower expression of PD-L1 (Fig. 2a, b). These results indicated a relatively lower response to immune checkpoint inhibitors in MM compared to CM, as was suggested in a previous study [53]. Interestingly, seven MM specimens and three MM specimens demonstrated higher tumor mutation burden or PD-L1 expression than CM, respectively (Fig. 2a, b, higher than median in CM cohort). The clinical relevance of high tumor mutation burden or PD-L1 expression in MM on immune checkpoint blockades still remains unknown, thus further investigation would reveal their potential as a predictor of response to immune checkpoint inhibitors.

Analyses on mutational spectrum and tumor mutation burden significantly differentiated primary anal MM from CM metastasized to the bowel. Although both melanoma types arise from melanocytes and grow in a similar mucosal microenvironment, the difference during their initiation, particularly the involvement of UV exposure, may lead to a distinct mutational landscape. Medical history or evidence of primary CM facilitates a definitive diagnosis between these types, however distinguishing these two types is occasionally challenging [45]. Targeted NGS potentially facilitates a definitive diagnosis of anal melanoma, leading to relevant therapies for either CM or MM.

## Conclusions

This study revealed potential mutagenic factor and driver mutations involved in MM. We identified *DCC* mutations as a potential prognostic marker in MM. Targeted NGS facilitates a definitive diagnosis of MM in anorectal regions. Although CM is highly associated with UV exposure and *BRAF*/*NRAS* mutations, low association to these signatures in MM strongly suggests that MM has distinct mechanisms involved in its initiation and progression, necessitating unique treatment strategies separate from CM treatment based on its molecular profile.

## Additional files

Additional file 1: Clinical characteristics of 89 melanomas. Description of data: Clinical information of each of the 89 individuals included in this study, including specimen name, gender, ethnicity, stage at tissue removal, age at tissue removal, sample type, tissue site, primary site, Breslow thickness in mm, and ulceration. (DOCX 31 kb)

Additional file 2: Gene List for Haloplex. Description of the genes targeted in the Haloplex pannel including, gene name, number of probes per gene, number of MM patients with a mutation in a given gene, number of CM patients with a mutation in a given gene. (DOCX 22 kb)

## Abbreviations

CM: Cutaneous melanoma
COSMIC: Catalogue of somatic mutations in cancer
CRC: Colorectal cancer
FFPE: Formalin-fixed paraffin-embedded
IGF: Insulin-like growth factor
IHC: Immunohistochemistry
JWCI: John Wayne Cancer Institute
MM: Mucosal melanoma
NGS: Next-generation sequencing
OS: Overall survival
SJHC: Saint John’s Health Center
TCGA: The Cancer Genome Atlas
Triple-WT: Triple wild-type
