# Supplementary material for: Predominance of triple wild-type and IGF2R mutations in mucosal melanomas
Source: BMC Cancer. 2018 Oct 30;18:1054. doi: 10.1186/s12885-018-4977-2 (PMC6206730; doi:10.1186/s12885-018-4977-2)
Supplement: Supplementary file 1 — Clinical characteristics of 89 melanomas. Description of data: Clinical information of each of the 89 individuals included in this study, including specimen name, gender, ethnicity, stage at tissue removal, age at tissue removal, sample type, tissue site, primary site, Breslow thickness in mm, and ulceration. (DOCX 31 kb) [file 12885_2018_4977_MOESM1_ESM.docx]

Table S1: Clinical characteristics of 89 melanomas

NA: not applicable, FFPE: formalin-fixed paraffin-embedded specimens

Gender: M (Male), F (Female)

Ethnicity: W (White), H (Hispanic), B (Black), A (Asian)

| Specimen | Gender | Ethnicity | Stage at tissue removal | Age at tissue removal | Sample type | Tissue site | Primary site | Breslow (mm) | Ulceration |
| --- | --- | --- | --- | --- | --- | --- | --- | --- | --- |
| Cutaneous 1 | F | NA | III | 59 | Frozen | Lymph node | Trunk | NA | NA |
| Cutaneous 2 | M | H | III | 40 | Frozen | Lymph node | Trunk | 2.15 | Yes |
| Cutaneous 3 | F | NA | III | 72 | Frozen | Lymph node | Extremity | 5.5 | Yes |
| Cutaneous 4 | M | W | III | 44 | Frozen | Lymph node | Extremity | 1.6 | NA |
| Cutaneous 5 | F | W | III | 30 | Frozen | Lymph node | Trunk | 1.4 | No |
| Cutaneous 6 | M | NA | III | 34 | Frozen | Lymph node | Trunk | 1.45 | No |
| Cutaneous 7 | F | W | III | 26 | Frozen | Lymph node | Trunk | 2.8 | No |
| Cutaneous 8 | F | W | III | 70 | Frozen | Lymph node | Trunk | 11 | Yes |
| Cutaneous 9 | M | W | III | 55 | Frozen | Lymph node | Head&Neck | 2.5 | No |
| Cutaneous 10 | M | W | III | 68 | Frozen | Lymph node | Extremity | 0.72 | No |
| Cutaneous 11 | M | W | III | 71 | Frozen | Lymph node | Trunk | 0.7 | NA |
| Cutaneous 12 | F | W | III | 73 | Frozen | Lymph node | Extremity | 0.85 | No |
| Cutaneous 13 | M | W | III | 48 | Frozen | Lymph node | Trunk | 3.5 | No |
| Cutaneous 14 | M | W | III | 44 | Frozen | Lymph node | Trunk | 10 | No |
| Cutaneous 15 | M | W | III | 52 | Frozen | Lymph node | Trunk | 2 | Yes |
| Cutaneous 16 | M | W | III | 67 | Frozen | Lymph node | Trunk | 2.08 | Yes |
| Cutaneous 17 | M | H | II or III | 54 | Frozen | NA | Head&Neck | 12.2 | No |
| Cutaneous 18 | M | W | III | 54 | Frozen | Lymph node | Trunk | 1.4 | No |
| Cutaneous 19 | M | W | III | 73 | Frozen | Lymph node | Extremity | NA | No |
| Cutaneous 20 | M | W | III | 47 | Frozen | Lymph node | Extremity | 5 | Yes |
| Cutaneous 21 | M | W | III | 46 | Frozen | Lymph node | Trunk | 1.35 | No |
| Cutaneous 22 | F | W | IV | 57 | Frozen | Adrenal Gland | Trunk | 0.8 | NA |
| Cutaneous 23 | M | W | IV | 47 | Frozen | Spleen | NA | NA | NA |
| Cutaneous 24 | M | W | IV | 54 | Frozen | Lung | Head&Neck | 1.4 | No |
| Cutaneous 25 | M | W | IV | 67 | Frozen | Lung | Head&Neck | 1.5 | Yes |
| Cutaneous 26 | M | W | IV | 37 | Frozen | Spleen | NA | NA | NA |
| Cutaneous 27 | M | W | IV | 54 | Frozen | Bowel | Trunk | 5 | NA |
| Cutaneous 28 | M | W | IV | 49 | Frozen | Lung | Head&Neck | 0.35 | No |
| Cutaneous 29 | M | W | IV | 68 | Frozen | Bowel | NA | NA | NA |
| Cutaneous 30 | M | W | IV | 32 | Frozen | Lung | Trunk | 0.7 | No |
| Cutaneous 31 | M | W | IV | 54 | Frozen | Bowel | Trunk | NA | NA |
| Cutaneous 32 | M | W | IV | 58 | Frozen | Lung | NA | NA | NA |
| Cutaneous 33 | M | W | IV | 68 | Frozen | Liver | NA | NA | NA |
| Cutaneous 34 | F | W | IV | 52 | Frozen | Spleen | Trunk | NA | No |
| Cutaneous 35 | M | W | IV | 52 | Frozen | Lung | Trunk | 2.1 | NA |
| Cutaneous 36 | F | W | IV | 47 | Frozen | Bowel | Extremity | 1 | No |
| Cutaneous 37 | M | W | IV | 71 | Frozen | Spleen | Trunk | 0.97 | No |
| Cutaneous 38 | M | W | IV | 60 | Frozen | Adrenal Gland | NA | NA | NA |
| Cutaneous 39 | M | W | IV | 41 | Frozen | Lung | Extremity | 1.9 | No |
| Cutaneous 40 | F | W | IV | 64 | Frozen | Lung | Extremity | 1.3 | Yes |
| Cutaneous 41 | M | W | IV | 41 | Frozen | Bowel | Extremity | 3.5 | No |
| Cutaneous 42 | F | W | IV | 53 | Frozen | Lung | Extremity | NA | NA |
| Cutaneous 43 | M | W | IV | 71 | Frozen | Adrenal Gland | Trunk | 2.9 | No |
| Mucosal 1 | F | A | IV | 44 | Frozen | NA | Genital | NA | NA |
| Mucosal 2 | F | H | IV | 30 | Frozen | Brain | Genital | NA | NA |
| Mucosal 3 | F | W | IV | 72 | Frozen | Local recurrence | Head&Neck | NA | NA |
| Mucosal 4 | F | W | III | 79 | Frozen | Lymph node | Head&Neck | NA | Yes |
| Mucosal 5 | M | NA | III | 38 | Frozen | Lymph node | Head&Neck | 4 | No |
| Mucosal 6 | F | W | IV | 67 | Frozen | NA | Anorectal | NA | No |
| Mucosal 7 | F | W | IV | 67 | Frozen | NA | Anorectal | NA | NA |
| Mucosal 8 | M | W | IV | 67 | Frozen | Local recurrence | Head&Neck | NA | NA |
| Mucosal 9 | M | W | IV | 36 | Frozen | Lymph node | Anorectal | 1.75 | No |
| Mucosal 10 | M | W | IV | 52 | Frozen | Bowel | Head&Neck | NA | Yes |
| Mucosal 11 | F | W | IV | 47 | Frozen | NA | Anorectal | 11 | NA |
| Mucosal 12 | F | W | IV | 55 | Frozen | NA | Anorectal | 2 | Yes |
| Mucosal 13 | F | W | III | 81 | Frozen | Lymph node | Anorectal | NA | NA |
| Mucosal 14 | F | W | III | 43 | Frozen | Local recurrence | Anorectal | NA | Yes |
| Mucosal 15 | F | W | IV | 48 | Frozen | NA | Head&Neck | NA | NA |
| Mucosal 16 | F | W | IV | 57 | Frozen | Local recurrence | Anorectal | 0.66 | No |
| Mucosal 17 | F | W | IV | 61 | Frozen | Lymph node | Genital | 17 | Yes |
| Mucosal 18 | F | B | III | 66 | Frozen | Lymph node | Genital | 4 | Yes |
| Mucosal 19 | F | W | I/II | 59 | Frozen | Primary | Head&Neck | NA | No |
| Mucosal 20 | F | W | III | 78 | FFPE | Primary | Genital | 1.2 | Yes |
| Mucosal 21 | F | W | III | 76 | FFPE | Primary | Anorectal | 1.1 | No |
| Mucosal 22 | M | W | III | 76 | FFPE | Lymph node | Anorectal | NA | No |
| Mucosal 23 | M | W | I/II | 57 | FFPE | Primary | Anorectal | NA | Yes |
| Mucosal 24 | F | W | I/II | 88 | FFPE | Primary | Genital | 1.3 | no |
| Mucosal 25 | F | B | I/II | 66 | FFPE | Primary | Genital | 15 | Yes |
| Mucosal 26 | F | W | IV | 85 | FFPE | Bowel | Genital | NA | NA |
| Mucosal 27 | F | W | I/II | 64 | FFPE | Primary | Anorectal | 1.2 | No |
| Mucosal 28 | F | W | III | 79 | FFPE | Lymph node | Anorectal | 6 | NA |
| Mucosal 29 | M | W | III | 49 | FFPE | Local recurrence | Head&Neck | NA | No |
| Mucosal 30 | M | W | I/II | 66 | FFPE | Primary | Head&Neck | NA | NA |
| Mucosal 31 | M | W | III | 88 | FFPE | Lymph node | Head&Neck | 1.25 | NA |
| Mucosal 32 | F | H | I/II | 71 | FFPE | Primary | Head&Neck | NA | NA |
| Mucosal 33 | F | W | III | 53 | FFPE | Primary | Genital | 2.8 | No |
| Mucosal 34 | M | W | NA | 71 | FFPE | Primary | Head&Neck | 20 | NA |
| Mucosal 35 | F | W | I/II | 67 | FFPE | Local recurrence | Head&Neck | NA | NA |
| Mucosal 36 | F | W | I/II | 75 | FFPE | Local recurrence | Head&Neck | NA | NA |
| Mucosal 37 | F | W | I/II | 89 | FFPE | Primary | Genital | NA | NA |
| Mucosal 38 | F | W | I/II | 53 | FFPE | Primary | Anorectal | <1.0 | No |
| Mucosal 39 | F | H | NA | 56 | FFPE | Local recurrence | Head&Neck | NA | NA |
| Mucosal 40 | M | W | I/II | 77 | FFPE | Primary | Head&Neck | NA | NA |
| Mucosal 41 | M | A | IV | 71 | FFPE | Superior vena cava | Head&Neck | NA | NA |
| Cutaneous 44 | F | W | IV | 50 | FFPE | Bowel | Extremity | NA | NA |
| Cutaneous 45 | M | W | IV | 59 | FFPE | Bowel | Extremity | 1.3 | no |
| Cutaneous 46 | M | W | IV | 43 | FFPE | Bowel | Trunk | 0.85 | No |
| Cutaneous 47 | M | W | IV | 55 | FFPE | Bowel | Head&Neck | 0.8 | NA |
| Cutaneous 48 | M | W | IV | 30 | FFPE | Bowel | NA | NA | NA |
