# Supplementary material for: Predominance of triple wild-type and IGF2R mutations in mucosal melanomas
Source: BMC Cancer. 2018 Oct 30;18:1054. doi: 10.1186/s12885-018-4977-2 (PMC6206730; doi:10.1186/s12885-018-4977-2)
Supplement: Supplementary file 2 — Gene List for Haloplex. Description of the genes targeted in the Haloplex pannel including, gene name, number of probes per gene, number of MM patients with a mutation in a given gene, number of CM patients with a mutation in a given gene. (DOCX 22 kb) [file 12885_2018_4977_MOESM2_ESM.docx]

Table S2: Gene list for Haloplex

89 genes comprising 2603 probes

Region Size: 774527bp

| Gene name | Number of probes | Number of MM patients with mutation | Number of CM patients with mutation |
| --- | --- | --- | --- |
| ACTL6A | 23 | 5 | 2 |
| ACTL6B | 16 | 1 | 4 |
| APC | 44 | 22 | 14 |
| ARID1A | 34 | 16 | 14 |
| ARID1B | 42 | 5 | 11 |
| ARID2 | 37 | 3 | 12 |
| ATM | 114 | 8 | 15 |
| ATR | 101 | 5 | 11 |
| AXIN2 | 20 | 1 | 4 |
| BAX | 7 | 0 | 1 |
| BRAF | 39 | 5 | 36 |
| BRCA1 | 50 | 3 | 5 |
| BRCA2 | 52 | 6 | 14 |
| CDC42 | 11 | 2 | 0 |
| CDH2 | 29 | 1 | 8 |
| CDK4 | 7 | 0 | 0 |
| CDK6 | 17 | 1 | 2 |
| CDK8 | 21 | 0 | 2 |
| CDKN2A | 7 | 1 | 6 |
| CDKN2B | 3 | 0 | 1 |
| CDKN2C | 3 | 0 | 3 |
| CTNNB1 | 17 | 2 | 3 |
| DCC | 70 | 5 | 34 |
| DKK1 | 4 | 2 | 1 |
| DKK2 | 13 | 0 | 5 |
| DKK3 | 11 | 0 | 0 |
| DNMT3A | 32 | 3 | 6 |
| DPF1 | 12 | 0 | 2 |
| EGFR | 58 | 5 | 5 |
| EPHA3 | 27 | 1 | 12 |
| EPHB6 | 15 | 0 | 6 |
| ERBB2 | 30 | 5 | 5 |
| ERBB3 | 25 | 3 | 3 |
| ESR1 | 15 | 3 | 4 |
| FAT4 | 39 | 8 | 58 |
| FBXW7 | 24 | 2 | 4 |
| FZD10 | 2 | 3 | 9 |
| HRAS | 3 | 1 | 1 |
| IDH1 | 16 | 0 | 3 |
| IDH2 | 13 | 2 | 0 |
| IGF1R | 38 | 4 | 11 |
| IGF2R | 80 | 18 | 9 |
| IRS2 | 3 | 2 | 14 |
| KIT | 34 | 4 | 2 |
| KRAS | 8 | 2 | 3 |
| LRP5 | 49 | 5 | 20 |
| LTK | 18 | 4 | 8 |
| MAP2K1 | 24 | 1 | 1 |
| MEN1 | 10 | 0 | 3 |
| MET | 38 | 5 | 19 |
| MLH1 | 40 | 1 | 5 |
| KMT2A | 71 | 14 | 16 |
| MSH2 | 40 | 4 | 6 |
| MTOR | 98 | 13 | 22 |
| MUTYH | 12 | 1 | 3 |
| MYC | 7 | 0 | 3 |
| NF1 | 106 | 9 | 19 |
| NF2 | 36 | 0 | 2 |
| NRAS | 8 | 7 | 15 |
| PIK3CA | 37 | 4 | 5 |
| PIK3CG | 19 | 4 | 10 |
| PMS1 | 31 | 2 | 4 |
| PPP6C | 15 | 2 | 4 |
| PTEN | 19 | 1 | 3 |
| RAC1 | 10 | 1 | 5 |
| RB1 | 50 | 2 | 3 |
| RET | 33 | 7 | 8 |
| RHOT1 | 50 | 0 | 5 |
| RUNX3 | 15 | 2 | 4 |
| SMAD2 | 18 | 4 | 0 |
| SMAD3 | 22 | 4 | 2 |
| SMAD4 | 23 | 3 | 2 |
| SMAD7 | 9 | 2 | 6 |
| SMARCA2 | 65 | 5 | 5 |
| SMARCA4 | 61 | 13 | 28 |
| SMARCD1 | 16 | 1 | 1 |
| SMARCD2 | 10 | 0 | 3 |
| SMARCD3 | 12 | 1 | 3 |
| SMARCE1 | 20 | 1 | 1 |
| SMARCB1 | 15 | 2 | 2 |
| SNX31 | 25 | 2 | 2 |
| STAT3 | 41 | 1 | 2 |
| STK19 | 10 | 1 | 4 |
| TCF7L2 | 29 | 4 | 3 |
| TET2 | 21 | 8 | 13 |
| TP53 | 15 | 4 | 9 |
| TSC2 | 62 | 12 | 20 |
| VHL | 8 | 3 | 5 |
| WT1 | 19 | 2 | 6 |
